# Local and Systemic Cytokine, Chemokine, and FGF Profile in Bacterial Chondronecrosis with Osteomyelitis (BCO)-Affected Broilers

**DOI:** 10.3390/cells10113174

**Published:** 2021-11-15

**Authors:** Alison Ramser, Elizabeth Greene, Robert Wideman, Sami Dridi

**Affiliations:** 1 Center of Excellence for Poultry Science, University of Arkansas, Fayetteville, AR 72701, USA; atramser@uark.edu (A.R.); esgreene@uark.edu (E.G.); rwideman@uark.edu (R.W.); 2 Department of Poultry Science, Cell and Molecular Biology Program, University of Arkansas, Fayetteville, AR 72701, USA

**Keywords:** cytokines, FGF, BCO, FHN, lameness, broilers

## Abstract

Complex disease states, like bacterial chondronecrosis with osteomyelitis (BCO), not only result in physiological symptoms, such as lameness, but also a complex systemic reaction involving immune and growth factor responses. For the modern broiler (meat-type) chickens, BCO is an animal welfare, production, and economic concern involving bacterial infection, inflammation, and bone attrition with a poorly defined etiology. It is, therefore, critical to define the key inflammatory and bone-related factors involved in BCO. In this study, the local bone and systemic blood profile of inflammatory modulators, cytokines, and chemokines was elucidated along with inflammasome and key FGF genes. BCO-affected bone showed increased expression of cytokines IL-1β, while BCO-affected blood expressed upregulated TNFα and IL-12. The chemokine profile revealed increased IL-8 expression in both BCO-affected bone and blood in addition to inflammasome NLRC5 being upregulated in circulation. The key FGF receptor, FGFR1, was significantly downregulated in BCO-affected bone. The exposure of two different bone cell types, hFOB and chicken primary chondrocytes, to plasma from BCO-affected birds, as well as recombinant TNFα, resulted in significantly decreased cell viability. These results demonstrate an expression of proinflammatory and bone-resorptive factors and their potential contribution to BCO etiology through their impact on bone cell viability. This unique profile could be used for improved non-invasive detection of BCO and provides potential targets for treatments.

## 1. Introduction

Modern broiler chickens are characterized by fast growth and high meat yield, resulting in a highly efficient means of producing animal protein. This achievement in performance has been met with physiological obstacles, such as lameness, which leads to animal welfare concerns and production loss [1]. A common cause of lameness is bacterial chondronecrosis with osteomyelitis (BCO), also known as femur head necrosis (FHN). BCO involves bacterial infection, bone attrition, and lack of healing in the proximal head of the femur and tibia [2]. These points are known to be key mechanical stress points within the skeletal system resulting in increased susceptibility to bacterial infection [3]. The highly vascularized avian growth plate coupled with the occurrence of mechanically induced “wound sites” within the highly dynamic network of cells of the growth plate creates the perfect storm for BCO [3]. The necrosis and attrition of bone with simultaneous bacterial infection leads to rapid deterioration of animal welfare, which is exacerbated by the inability to detect BCO without necropsy, especially when subclinical. While it is clear, that BCO-affected broilers are under multiple stressors, both immune and physiological, it has yet to be investigated whether BCO has systemic effects on inflammatory pathways that may contribute to the etiology seen locally, which could also be used as a means of detection via a circulating biomarker.

The key characteristics of BCO, infection, inflammation, and lack of healing or regeneration provide a roadmap of the potential pathways involved. As mediators of the inflammatory response under both stress and bacterial infections, cytokines and chemokines have been implicated in numerous skeletal diseases including rheumatoid arthritis, osteoarthritis, periodontitis, and osteomyelitis [4,5,6,7,8,9]. Their involvement in inflammasome activation has also been well documented and links their roles to cell survival and homeostasis [10]. Additionally, these molecules have key physiological functions in bone development and homeostasis [11,12]. Fibroblast growth factors regulate healing and development as well as immune cell states and function in both bone and on a systemic level [13,14]. Specifically, FGF23 is a key factor in the bone–kidney–cardiac–immune axis as well as macrophage transition under inflammatory states [15]. FGF pathways have also been implicated in healing and bone regeneration, which is a major tenant lacking in BCO-affected bone [13]. Therefore, this study was undertaken to investigate whether systemic effects of BCO exist as well as profile the local and systemic expression of cyto(chemo)kines and key FGFs and evaluate their potential role in BCO etiology.

## 2. Materials and Methods

### 2.1. Bone and Blood Sample Collection

All animal experiments were approved by the University of Arkansas (Fayetteville, AR) Animal Care and Use Committee (protocol number 15043) and were in accordance with recommendations in *NIH’s Guide for the Care and Use of Laboratory Animals*. The BCO model and healthy counterparts were conducted as previously described [16]. Briefly, animals were placed on either litter or wire-flooring and had ad libitum access to fresh water and feed for 56 days. The wire-flooring environment consisted of a raised floor made of wire-covered panels to create unsteady footing. This design has been shown to induce spontaneous BCO in broilers in addition to increasing the occurrence of lameness [16]. Ambient temperature and lighting conditions followed industry standards, as previously described [10]. At the end of the 56 days, animals were assessed for the presence or absence of lameness. First, blood was drawn from the wing vein of lame birds that were reared on wire-flooring and non-lame birds reared on litter. Next, birds were humanely euthanized and immediately necropsied to determine presence of subclinical lesions in the proximal heads of both the femora and tibiae. Bone was selected macroscopically based on a previously reported scale [16]. Normal blood and bone came from non-lame birds, reared on litter that did not have BCO lesions. BCO-affected blood and bone were from lame birds reared on wire flooring with severe BCO lesions in both legs. Proximal portions of bone, primarily consisting of the growth plate, were snap frozen in liquid nitrogen and stored at −80 °C for later analysis. Blood was either stored in heparin-treated tubes for cell treatment or Trizol reagent (Life Technologies, Carlsbad, CA, USA) for RNA isolation.

### 2.2. Cell Culture and Treatment

Human fetal osteoblast (hFOB) 1.19 cells (CRL-11372; ATCC, Manassas, VA, USA) were cultured in a 1:1 mixture of Ham’s F12 medium/Dulbecco’s modified Eagle’s medium (DMEM), 10% fetal bovine serum (FBS), and 0.3 mg/mL G418. Cells were grown at 34 °C in a humidified atmosphere of 95% air and 5% CO_2_. Chicken primary chondrocytes were grown in a media of DMEM with 4.5 g glucose/L, glutamine, HEPES, and sodium pyruvate supplemented with 10 ng/mL ascorbic acid, 10% FBS, and 1% penicillin/streptomycin. Primary chondrocyte cells were grown at 37 °C in a humidified atmosphere of 95% air and 5% CO_2_. At 80% confluence, cells were synchronized using serum-free media overnight before being treated with human recombinant IL-1β (100 ng/mL) (PreproTech, East Windsor, NJ, USA), IL-8 (100 ng/mL) (R&D Systems, Minneapolis, MN, USA), TNFα (10 ng/mL) (PreproTech, East Windsor, NJ, USA), or dimethyl sulfoxide (DMSO) as the control for 24 h (*n* = 3/group). Following treatment, cells were processed for viability as described below. Additionally, hFOB cells were washed three times with phosphate-buffered saline (PBS) and treated with media containing either 5% plasma from healthy or BCO-affected birds or PBS (*n* = 3/group). Cells were maintained for an additional 4 h before being processed for cell viability.

### 2.3. Cell Viability

Cell viability for hFOB cells was performed as previously described [17]. Briefly, cells were seeded at 1 × 10^4^ cells per well of a 96-well plate before being treated as described above. CellTiter 96 AQueous One Solution CellProliferation Assay (Promega, Madison, WI, USA) was used, according to the manufacturer’s recommendations, and results were obtained using a Synergy HT multimode microplate reader (BioTek, Winooski, VT, USA). All sample readings were background corrected, and results were reported relative to the control.

Cell viability for primary chondrocyte cells was conducted using Trypan Blue live-dead stain (0.4%) (Life Technologies, Carlsbad, CA, USA) and quantified using Countess II FL (Life Technologies, Carlsbad, CA, USA).

### 2.4. RNA Isolation, Reverse Transcription, and Real-Time Quantitative PCR

From the normal and BCO-affected bone and blood samples (*n* = 4/group), total RNA was isolated in accordance with the protocol of previous work [18]. Total RNA was isolated using Trizol reagent (Life Technologies, Carlsbad, CA, USA), based on the manufacturer’s instructions. RNA concentrations were determined using a Synergy HT multimode microplate reader and total RNA was reverse transcribed using qScript cDNA SuperMix (Quanta Biosciences, Gaithsburg, MD, USA). Amplification was achieved using Power SYBRGreen Master Mix (Life Technologies, Carlsbad, CA, USA) and real-time quantitative PCR (7500 Real Time System; Applied Biosystems, Foster City, CA, USA). The sequences for oligonucleotide primers for *r18s*, *TNFα*, *IL-1β*, *IL-18*, *IL-3*, *IL-4*, *IL-10*, *IL-6*, *CRP*, *CCLL-4*, *CXCL-14*, *CCL-4*, *CCL-20*, *NLRP3*, *NLRC5*, *NLRX1*, and *NLRC3* were previously published [10,19,20]. Additional primers used were *IL-17* (forward, 5′-CCTTGCTCCTGCTTGCTTTC-3′; and reverse, 5′-AACCAATCGCTCCCCATTTT-3′), *IL-12B* (forward 5′-TGCCCAGTGCCAGAAGGA-3′; and reverse, 5′-TCAGTCGGCTGGTGCTCTT-3′), and chemokines *IL-8L1* (forward 5′-CAGAACCAAACCCAGGTGACA-3′; and reverse, 5′-ACAGCCTTGCCCATCATCTT-3′), *IL-8L2* (forward 5′-TCCTGGTTTCAGCTGCTCTGT-3′; and reverse, 5′-CGCAGCTCATTCCCCATCT-3′), *CCL-5* (forward 5′-TTTCTACACCAGCAGCAAATGC-3′; and reverse, 5′-GCCCCTTCCTGGTGATGAA-3′). Also measured were *FGF23* (forward 5′-CTGCTTGTGCTCTGTATCCTGAA-3′; and reverse, 5′-CAGCAGCGGAGAGGAGTTG-3′), *FGFR1* (forward 5′-GCCCCGGAGGCTCTGT-3′; and reverse, 5′-CCGAAGGACCAAACATCACTCT-3′), and *Klotho* (forward 5′-TGGCGATGTCCCGGTTTAT-3′; and reverse, 5′-ATATACTCTGAGCTTATCGTGCACCAT-3′).

Real-time quantitative PCR cycling conditions were 50 °C for 2 min, 95 °C for 10 min, and 40 cycles of a two-step amplification (95 °C for 15 s followed by 58 °C for 1 min). The dissociation protocol from the sequence detection system was used for melting curve analysis to exclude potential contamination of non-specific PCR products. Negative controls that were used as templates contained no reverse transcription products. Relative expression of target genes was determined using the 2^−∆∆CT^ method and healthy bone tissue or untreated cells were used as calibrators [21].

### 2.5. Statistical Analysis

Data were analyzed by Student *t*-test or one-way ANOVA, as appropriate, using GraphPad version 7.03 (GraphPad Software, Inc., LaJolla, CA, USA). Results are expressed as means ± SEM, with *p*-value < 0.05 set as statistically significant.

## 3. Results

### 3.1. Plasma from BCO-Affected Broilers Significantly Decreased hFOB Cell Viability

**Figure 1:**
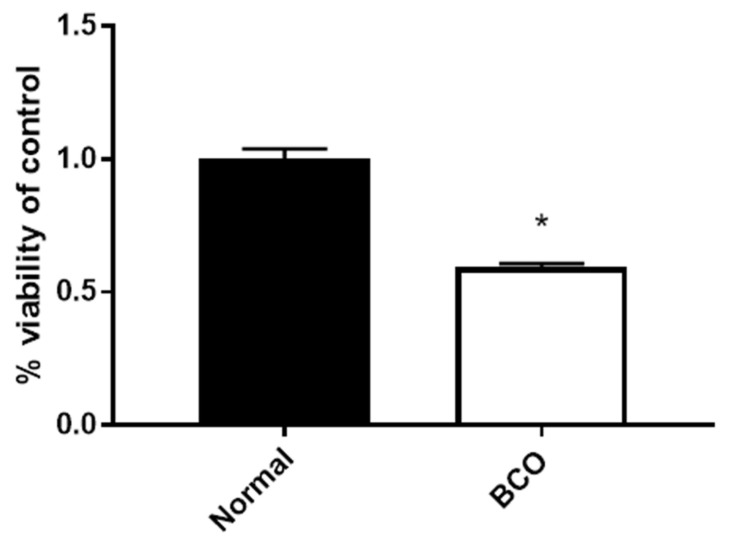
Effect of plasma from normal and BCO-affected broilers on hFOB cell viability. MTT assay results from hFOB cells treated with media containing 5% plasma from either normal (non-lame and no lesions of the bone) or BCO-affected (lame with lesions in both legs) broilers for 4 h. Significance was determined using a student *t*-test with *p*-value < 0.05. * indicates a significant difference between groups.

hFOB cells were exposed to fresh plasma from birds both lame and affected by BCO as well as plasma from non-lame and apparently normal bone for 4 h. Cell viability was significantly decreased in cells exposed to plasma from BCO-affected birds when compared to normal plasma (Figure 1) (*p* < 0.05).

### 3.2. Unique Expression Profile of Inflammatory and Bone-Related Cytokines and Chemokines in the Bone and Blood of BCO-Affected Broilers

**Figure 2:**
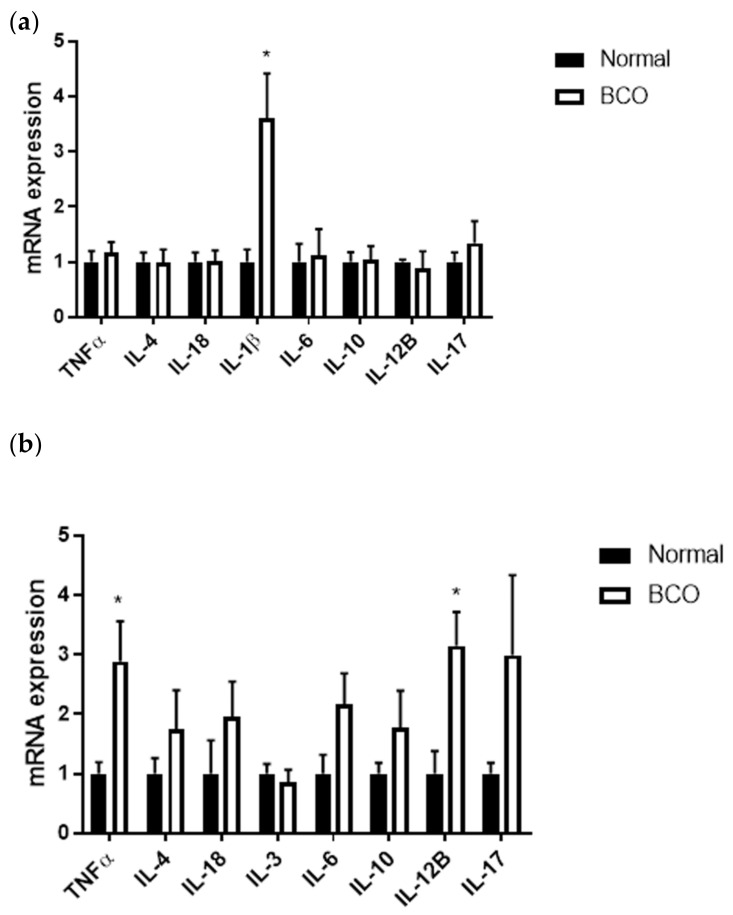
Local and systemic cytokine profile in normal and BCO-affected broilers. Gene expression for *TNFα*, *IL-4*, *IL-18*, *IL-1β*, *IL-6*, *IL-10*, *IL-12B*, *IL-17,* and *IL-3* in bone (**a**) and blood (**b**) of normal and BCO-affected broiler chickens. Significance was determined using a student *t*-test with *p*-value < 0.05. * indicates significant difference between normal and BCO.

The local cytokine profile was determined through mRNA expression in normal and BCO-affected bone. Only one significant difference was seen between BCO and normal bone, with *IL-1β* being significantly upregulated in BCO by approximately four times the fold change (Figure 2a) (*p* < 0.01). The systemic cytokine profile was determined by determining the gene expression of cytokines within the whole blood and showed significant upregulation of *TNFα* and *IL-12B* (Figure 2b) (*p* < 0.05). In both bone and blood, expression of *IL-4*, *IL-18*, *IL-6*, *IL-10*, and *IL-17* was not significantly affected (Figure 2a,b). In blood, *IL-3* was also measured, but its expression remained similar between normal and BCO (Figure 2b).

**Figure 3:**
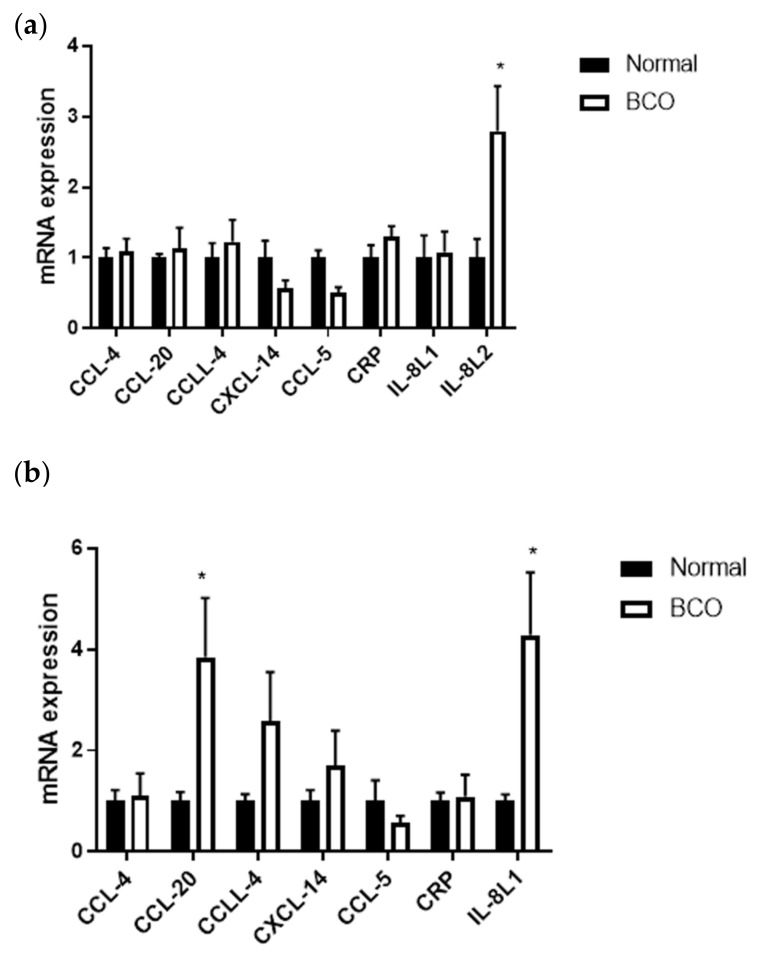
Local and systemic chemokine profile in normal and BCO-affected broilers. Gene expression for *CCL-4*, *CCL-20*, *CCLL-4*, *CXCL-14*, *CCL-5*, *CRP*, and *IL-8L1*/*IL-8L2* in bone (**a**) and blood (**b**) of normal and BCO-affected broiler chickens. Significance was determined using a student *t*-test with *p*-value < 0.05. * indicates significant difference between normal and BCO.

The chemokine profile was also evaluated in the bone and blood of BCO-affected and normal birds. In bone, *IL-8L2* was significantly upregulated in BCO compared to bone without lesions (Figure 3a) (*p* < 0.05). While expression of *IL-8L1* was not significantly different in bone, *IL-8L1* was significantly upregulated in the blood of BCO-affected birds compared to the normal group (Figure 3b) (*p* < 0.05). Additionally, the chemokine *CCL-20* was significantly upregulated in blood but not locally in the bone. *CCL-4*, *CCLL-4*, *CXCL-14*, *CCL-5*, and *CRP* expression was not significantly different in bone nor blood (Figure 3a,b) (*p* > 0.05).

### 3.3. Circulating Levels of Inflammasome NLRC5 Were Significantly Increased in BCO

**Figure 4:**
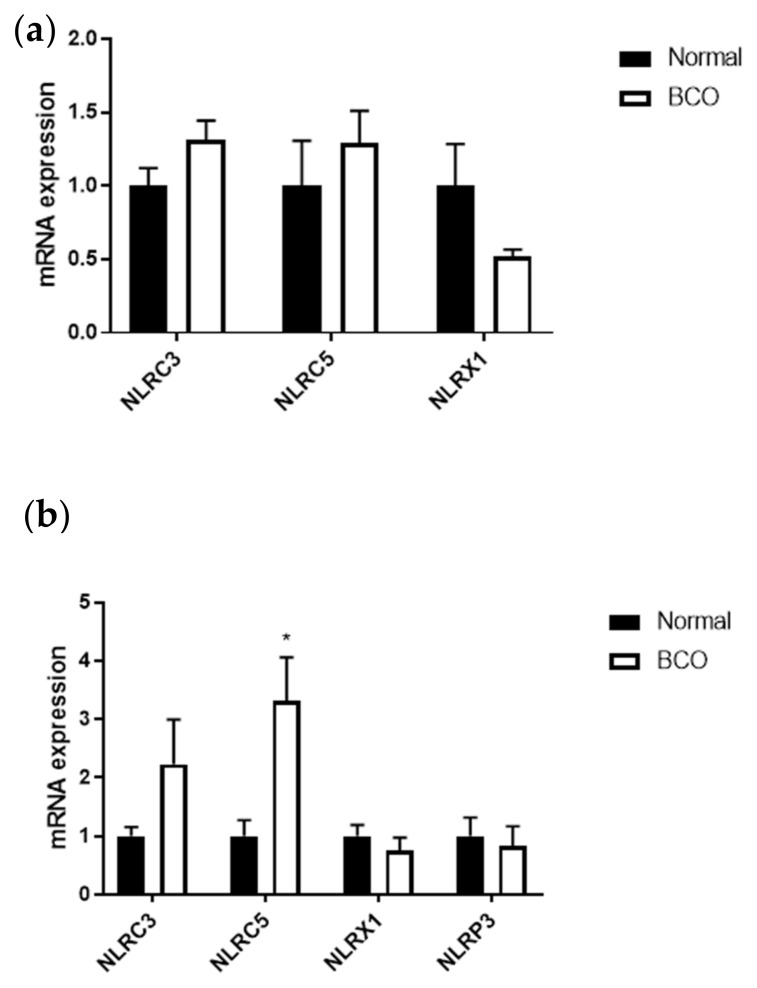
Local and systemic inflammasome profile in normal and BCO-affected broilers. Gene expression for *NLRC3*, *NLRC5*, and *NLRX1* in bone (**a**) and blood (**b**) of normal and BCO-affected broiler chickens, as well as the expression of NLRP3 in blood (**b**). Significance was determined using a student *t*-test with *p*-value < 0.05. * indicates a significant difference between normal and BCO.

While there were no significant differences in inflammasome expression in bone, blood from BCO-affected birds had significantly higher *NLRC5* mRNA expression compared to normal birds (Figure 4a,b) (*p* > 0.05; *p* < 0.05).

### 3.4. Key Receptor in the FGF23 Pathway was Significantly Downregulated in BCO-Affected Bone

**Figure 5:**
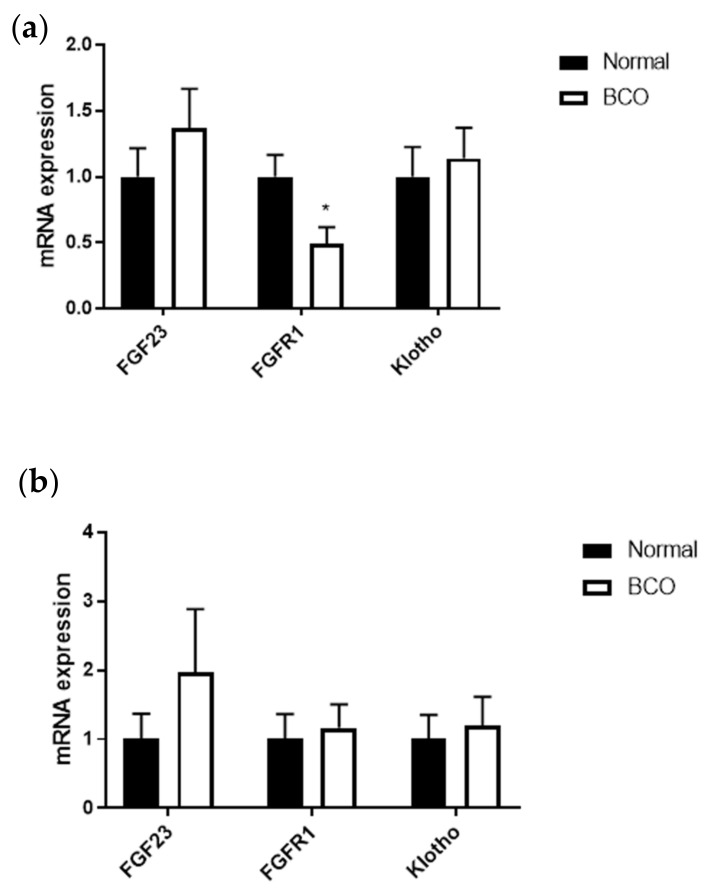
Local and systemic expression of key FGFs in normal and BCO-affected broilers. Gene expression for *FGF23*, *FGFR1*, and *Klotho* in the bone (**a**) and blood (**b**) of normal and BCO-affected broiler chickens. Significance was determined using a student *t*-test with *p*-value < 0.05. * indicates a significant difference between normal and BCO.

In the local bone, *FGFR1* expression was significantly decreased in BCO-affected tissue (Figure 5a) (*p* < 0.05). No significant differences were seen in the blood between the two groups (Figure 5b).

### 3.5. Recombinant IL-1β and TNFα Proteins Significantly Decreased Cell Viability in Both Human and Chicken Bone Cells

**Figure 6:**
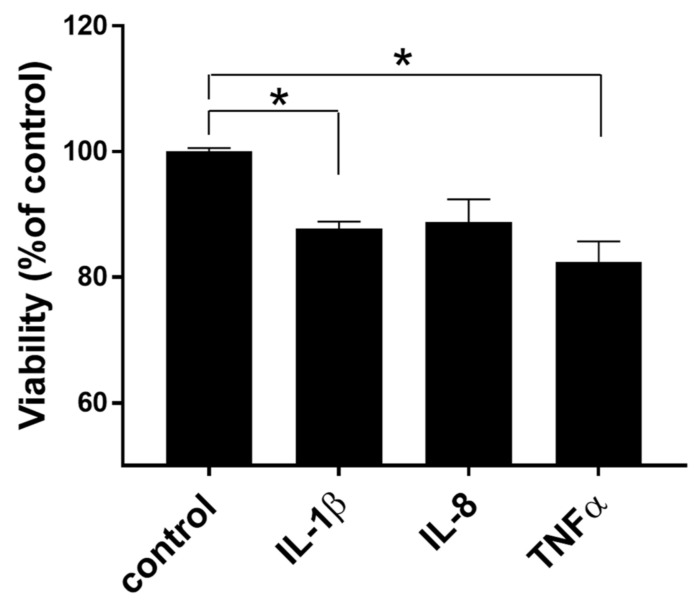
Effect of human recombinant IL-1β, IL-8, and TNFα on hFOB cell viability MTT assay results from hFOB cells treated with human recombinant IL-1β (100 ng/mL), IL-8 (100 ng/mL), and TNFα (10 ng/mL) for 24 h. Significance was determined using a student *t*-test with *p*-value < 0.05. * indicates a significant difference between treated groups and the control.

Treatment with human recombinant IL-1β or TNFα significantly reduced cell viability in hFOB cells by approximately 18% (*p* = 0.0036 for IL-1β and *p* = 0.0249 for TNFα, Figure 6). Similar results were seen in chicken primary chondrocyte cells (data not shown).

## 4. Discussion

Modern medical research has revealed intense networks of molecular signaling and action under disease and infectious states with far-reaching systemic impacts. In BCO, the suspected delivery of bacteria to the bone via the high vascularization in the growth plate coupled with the evidence of chronic inflammation and lack of healing could drive systemic effects within the broiler detectable within circulation. If true, this could act as a non-invasive biomarker for BCO. Thus, the potential systemic effects in BCO were first investigated via exposure of hFOB cells to plasma from normal and BCO-affected birds. The resulting decreased cell viability points to potential circulating factors that affect bone cellular function and viability.

Under an inflammatory and infectious state, such as BCO, cyto(chemo)kines play an integral role via both their local and systemic action and stimuli [22]. Specifically, cytokines are cell signaling molecules, which drive inflammation via their effects on immune cell activation and modulation as well as downstream effects within a multitude of cell types [23]. In BCO-affected bone, there was a clear increase in interleukin-1β (IL-1β) mRNA expression while other cytokines measured were unaffected. This corroborates previous work done in BCO, which showed that increased IL-1β expression was coupled with dysregulation of Dicer 1 Ribonuclease III and leucine-rich containing protein 3 (NLRP3) inflammasome activation [10]. IL-1β is a well-studied proinflammatory cytokine with roles not only in inflammasome activation and apoptotic pathways [23,24]. Additionally, both IL-1β and TNFα have been shown to decrease chondrocyte proliferation and hypertrophy, necessary steps in endochondral ossification and proper growth plate homeostasis [25]. In osteoarthritis, IL-1β expression was shown to increase in conjunction with decreased extracellular matrix formation by chondrocytes contributing to osteoarthritic cartilage degradation [26]. Serum levels of IL-1β were increased in broilers with femur head necrosis in an experimental model involving methylprednisolone intramuscular injections, which also coincided with disruption of extracellular matrix homeostasis [27]. These results support the finding of increased expression of IL-1β in BCO-affected bone in this study. In BCO, IL-1β could be acting not only as a proinflammatory influencer, but as a contributor to decreased cartilage stability and maturation, increasing susceptibility to mechanical stress and bacterial infection.

Cytokines upregulated in circulation included tumor necrosis factor α (TNFα) and IL-12B. Like IL-1β, TNFα is a well-studied potent proinflammatory cytokine of the TNF super family. It acts via induction of cytokine production, stimulation of growth, and activation of adhesion molecules [28,29,30]. Additionally, TNFα has roles in metabolism, such as insulin resistance and lipid metabolism as well as skeletal growth [30]. Within bone, TNFα has been shown to induce osteoclastogenesis, thereby impacting the ratio of bone-resorbing to bone-mineralizing cells. Indeed, overexpression of TNFα in mice resulted in growth retardation resulting from decreased insulin-like growth factor 1 (IGF1) and IGF1-binding protein (IGFBP3) [31]. This, coupled with its effects on chondrocytes previously described, could have effects on bone healing and growth plate integrity under BCO conditions. It is well documented that bone healing is driven through a shift from bone-resorbing pathways and cell types, such as osteoclasts, to bone-mineralizing pathways and cell types, such as osteoblasts. Interestingly, surface proteins of *Staphylococcus aureus*, one of the most common causative agents in BCO, have been shown to induce bone loss via potent osteolytic effects. These effects were inhibited by IL-1 receptors and TNFα antibodies [32]. Both IL-1β and TNFα act via catabolic mechanisms within bone and proinflammatory mechanisms systemically, which could be contributing to the lack of healing seen in BCO. Other studies investigating these cytokines in osteomyelitis found increased TNFα in the early stage of chronic osteomyelitis rather than the late stage [33]. Another study involving the stimulation of blood from patients with osteomyelitis with lipid polysaccharides (LPS) did not affect the expression of TNFα or other cytokines in the blood [34].

While the cytokines implicated thus far have been proinflammatory, IL-12B is a primarily anti-inflammatory cytokine [35]. Anti-inflammatory cytokines act by decreasing proinflammatory cytokine production and changing immune cell activation or expression, such as the T helper 1 and T helper 2 profiles [36,37,38,39,40]. However, in the case of IL-12B, the combination of cytokine ligand and receptor, as well as the physiological conditions, determines whether its function is anti- or proinflammatory [35,37]. In the case of BCO, further investigation into cytokine receptor expression as well as protein expression is necessary to fully elucidate the role of IL-12B in circulation.

Another key molecule in inflammatory states that has been implicated in disease states are chemokines. Chemokines act by directing the circulation of lymphoid cells and recruiting them under conditions, such as injury and infection [41,42]. Nomenclature for chemokines involves denoting the spacing of the first two cysteines on the amino acid terminals of the proteins with either CC, CXC, XC, or CX3C followed by “L” to indicate it is the ligand [23]. CCL-20 was significantly upregulated in the blood of BCO-affected broilers and it has been shown to be induced by TNFα in several cell types [23]. It is primarily a homeostatic chemokine with constitutive expression in several tissues. However, it has been shown that CCL-20 plays a role in the recruitment of immature dendritic cells and precursors to sites of infection and injury and that CCL-20 is involved in pathologies of the endothelial surfaces, such as inflammatory bowel disease and rheumatoid arthritis [43,44,45]. In rheumatoid arthritis, CCL-20 expression correlated to bone loss and patient survival [46]. Its presence in the circulation of BCO-affected broilers could be due to the simultaneous increased expression of TNFα leading to downstream effects in both immune cell recruitment and bone loss.

In both the bone and blood of BCO-affected birds, IL-8 was increased. Granted IL-8L2 was increased in bone and IL-8L1 was increased in blood, the two avian orthologs have been shown to have similar functions and comparable homology to human CXCL8 [47,48]. IL-8 acts primarily in the activation and migration of monocytes, lymphocytes, and other immune cells to sites of infection and injury [49]. It is considered a key inflammatory mediator and shown to have angiogenic effects [49,50]. In bone, IL-8 expression by osteoblasts and osteoclasts has been shown to be induced by cytokines IL-1β and TNFα, both shown here to be upregulated in BCO [51,52]. IL-8 has also been shown to trigger circulating monocytes to undergo osteoclast formation [53]. Notably, breast cancer patients with bone metastases were shown to have elevated levels of plasma IL-8, which also correlated with increased bone resorption [54]. The results of this study corroborate with these studies and suggest further evidence of an imbalance in bone resorption to bone-forming pathways in BCO.

Given the clear upregulation of certain cyto(chemo)kines both in local bone and systemic blood circulation of BCO-affected broilers, the inflammasome profile was also evaluated. NLRP3 has already been shown to be increased in BCO bone [10]. Results showed that NLRC5 was also significantly increased in the blood of BCO-affected broilers. NLRC5 has been shown to have potential influences on the nuclear factor kappa B (NF-κB) pathway as well as NLRP3 inflammasome activation by working in the complex-forming step [55]. Inflammasome complexes are a part of the innate immune system initiating inflammation. Their activation leads to the activation of caspase-1, which is responsible for the cleavage and subsequent activation of proinflammatory cytokines, such as IL-1β [56]. Its expression has been seen in the primary cells of myeloid and lymphoid origin as well as bone marrow and B cells and to be upregulated in chicken macrophages under inflammatory states [57,58]. Interestingly, NLRC5 is seen to play an active role in the first stage of inflammation and a diminished role with the process of inflammation [59]. The upregulation of blood NLRC5 expression in BCO-affected birds could indicate its involvement in the induction of the other proinflammatory cytokines that are upregulated and as an indicator that BCO represents a persistent inflammatory state.

In addition to inflammasome complexes, cyto(chemo)kines have influences on and are affected by FGFs [60]. FGFs have far-reaching effects on both immune response and healing or homeostasis of bone [13,61]. Indeed, the role of FGF signaling in skeletal formation has been demonstrated via gain-of-function mutations in the FGF receptor (FGFR) genes [62]. Expressions of key FGFs involved in bone processes as well as the healing and immune response were evaluated. Notably, FGFR1, which has a diverse and expansive repertoire of ligands and effects in both bone and immune responses, was significantly downregulated in BCO-affected bone. FGFR1 is the receptor for FGF23, which is primarily produced by the bone and involved in immune and homeostasis functions [14]. FGFR1 knockout mice showed no difference to the control in bone mineral density or soft X-ray imaging of the femur; however, the lack of FGFR1 in the bone coincided with decreased serum phosphate, serum FGF23, and expression of FGF23 within the bone in addition to decreased body weight and a shorter lifespan [15]. Extracellular phosphate is involved in the induction of processes necessary within hypertrophic chondrocytes for normal endochondral bone development as well as the development of vascular diseases [63,64]. Therefore, a lack of FGFR1 could be contributing to a loss of bone integrity by disrupting hypertrophic chondrocyte function. Further research into the protein expression and modification state of FGFR within BCO-affected bone is needed to fully understand what its downregulation could mean for BCO etiology. However, this is the first time the FGF pathway has been implicated in BCO in modern broilers and warrants further investigation.

Given the clear profile of proinflammatory cytokines and chemokines in BCO, recombinant proteins were used to determine if these could be the factors within BCO plasma responsible for the decreased cell viability in hFOB cells. To this end, both hFOB cells and chicken primary chondrocyte cells were challenged with IL-1β, IL-8, and TNFα. Of the three treatments, TNFα significantly reduced cell viability in both cell types. Decreased viable osteoblast-type cells due to the circulating levels of TNFα could be contributing to the etiology of BCO by shifting the ratio of bone-resorbing and bone-forming cells. Additionally, decreased viability of chondrocytes within the growth plate could contribute to its susceptibility to infection and mechanical stress. However, it is unclear if the TNFα expression is the result of or a contributor to the bacterial infection and bone loss. An essential piece missing in the understanding of BCO is its progression at both a cellular and systemic level and warrants further research. Identifying the culpable circulating cell types is also warranted for future research as it could elucidate the means in which BCO exerts systemic effects.

## 5. Conclusions

In this study, plasma from BCO-affected broilers had significant effects on the viability of hFOB cells, demonstrating circulating factors that influence bone cell function and survival. This is the first study profiling cyto(chemo)kines and key FGFs in BCO bone and blood and demonstrating that elevated TNFα expression in the blood of BCO-affected birds could be the causative agent in bone cell viability. The unique circulating profile of BCO-affected birds provides a potential means of non-invasive detection and potential targets for treatment. Additionally, this is the first study to investigate FGFs in relation to BCO and showed decreased FGFR1 in the BCO-affected bone. Taken together, these results indicate increased expression of proinflammatory and bone-resorptive factors, which could be contributing to the bone loss and necrosis seen in BCO. Further research is needed to elucidate the direct mechanisms behind these factors and their effects.

## Data Availability

All data are included in the paper.
